# Temporal Trend and Fluctuation Learning via Enhanced Attention Mamba for Carbon Price Interval Forecasting

**DOI:** 10.3390/e28030270

**Published:** 2026-02-28

**Authors:** Lijun Duan, Jin Chen, Qiankun Zuo, Yanfei Zhu, Yi Di, Ruiheng Li

**Affiliations:** 1 School of Computer Science and Artificial Intelligence, Hubei University of Education, Wuhan 430205, China; duanlijun@hue.edu.cn; 2 Hubei Key Laboratory of Digital Finance Innovation, Hubei University of Economics, Wuhan 430205, Chinaliruiheng@hbue.edu.cn (R.L.); 3 School of Computer Science, Hubei University, Wuhan 430062, China; 4 School of Information Engineering, Hubei University of Economics, Wuhan 430205, China; 5 College of Education Science, Hubei Normal University, Huangshi 435002, China; yafeizhu@foxmail.com

**Keywords:** carbon price forecasting, bidirectional state space models, interval-valued recovery loss, wavelet decomposition, trading market

## Abstract

Accurate carbon price forecasting is essential for transforming complex carbon trading markets into efficiently managed and stably operating systems. Existing long-term time series forecasting methods struggle to capture the nonlinear and non-stationary characteristics inherent in carbon prices. To address this limitation, we propose the Temporal Trend and Fluctuation Learning (TTFL) model for interval-valued carbon price forecasting. The model first uses wavelet decomposition to separate the forecasting task into two branches: Price Trend Learning (PTL) and Price Fluctuation Learning (PFL). The PTL branch adopts a forward–backward enhanced Mamba architecture to extract low-frequency, long-term trend features. This design facilitates price interactions across time steps. The enhanced Mamba module leverages a state space model (SSM) to preserve historical information selectively and employs a forgetting gate to recover missing information. As a result, the model captures complementary dependencies across different price points, improving prediction reliability. The PFL branch integrates an attention mechanism with the standard Mamba architecture to model high-frequency temporal dynamics. It provides fine-grained short-term volatility information essential for market participants. We also introduce an interval-valued recovery loss function. This loss quantifies the overlap between predicted and actual interval prices, emphasizes trend learning, and stabilizes model training. We evaluate the TTFL model on three real-world carbon trading markets. Comparative experiments demonstrate that TTFL achieves superior prediction accuracy and robustness relative to baseline methods. Through collaborative learning and selective state space modeling, our approach not only outperforms traditional forecasting models but also offers stakeholders a practical tool for navigating complex carbon market environments. This work contributes a novel forecasting paradigm that integrates multivariate collaborative learning with selective state space modeling. It provides actionable insights for policymaking, investment strategy development, and risk management in the energy and environmental sectors.

## 1. Introduction

Global consensus on climate change and environmental protection has strengthened considerably. Reducing greenhouse gas emissions—particularly carbon dioxide (CO_2_)—now represents a major challenge confronting governments, enterprises, and international organizations [1,2]. To address this challenge effectively, carbon pricing mechanisms have emerged. They have become a key policy instrument for advancing low-carbon transition worldwide. By assigning an economic cost to carbon emissions, these mechanisms incentivize emission reductions in production and consumption. They also drive the development and deployment of low-carbon technologies and clean energy. Carbon trading markets have now been established in many countries and regions. Within these markets, carbon price serves as a fundamental indicator of emission costs and supply–demand dynamics. It directly affects enterprises’ production costs and profitability [3,4,5]. Moreover, carbon price fluctuations profoundly influence the global energy mix, industrial structure, and broader economic development pathways. Accurate carbon price forecasting has therefore become a critical challenge for policymakers, market participants, and academic researchers alike.

Carbon price forecasting seeks to integrate diverse data sources—including historical prices, macroeconomic indicators, and policy developments—with advanced methodologies from econometrics, statistics, and machine learning. The objective is to uncover the underlying patterns and drivers of carbon price fluctuations and to generate accurate predictions of future price trends [6,7]. Researchers have explored a range of methodological frameworks for point forecasting of carbon prices based on historical and economic data. Early empirical models, such as autoregressive approaches, were widely adopted in financial time-series prediction due to their ability to capture periodic and regular patterns [8,9]. For instance, Arouri et al. [10] employed an autoregressive model to investigate the dynamic relationship between spot and future prices of European Union Emission Allowances (EUAs) during the second trading phase of the EU ETS. García-Martos et al. [11] extended the autoregressive framework to forecast multiple interrelated price series—including fossil fuels, CO_2_ allowances, and electricity—thereby offering a broader decision-support tool. However, the carbon market is characterized by instability and strong nonlinearity. Under these conditions, traditional empirical models struggle to deliver accurate carbon price forecasts.

Advanced machine learning techniques are now widely adopted for carbon price forecasting due to their strong capability in nonlinear time-series modeling [12,13,14]. For instance, Li et al. [15] employed a back-propagation neural network to predict long-term carbon prices under six energy-related scenarios. Xu et al. [16] introduced an Extreme Learning Machine (ELM) to capture complex temporal dynamics. Their model effectively handles carbon price series exhibiting multiple frequencies. To address the inherent instability of carbon prices, researchers have developed hybrid frameworks that integrate multiple advanced machine learning techniques. These approaches aim to mitigate noise interference and enhance forecasting accuracy. Huang et al. [17] proposed a hybrid model based on Variational Mode Decomposition (VMD). First, VMD decomposes the original carbon price series into several components. Then, Long Short-Term Memory (LSTM) and Generalized Autoregressive Conditional Heteroskedasticity (GARCH) models are respectively employed to learn low-frequency and high-frequency dynamics. Finally, all components are ensembled to generate the final forecast. Similarly, Zhang et al. [18] combined VMD, LSTM, and Support Vector Regression (SVR) for coal price forecasting. Their results demonstrate that such hybrid architectures can leverage the strengths of individual components to produce accurate and robust predictions.

Carbon prices in emissions trading markets are influenced by multiple interrelated factors, including macroeconomic conditions, energy prices, regulatory policies, and climate events, among others. The inherent uncertainty of these drivers makes it difficult for point forecasting methods to accurately capture future price fluctuations. Interval-valued forecasting offers a compelling alternative. It predicts the plausible fluctuation range of future carbon prices rather than a single value. By explicitly accounting for uncertainty, interval-valued approaches provide more comprehensive and informative market signals [19,20,21,22,23]. Recent studies have increasingly focused on interval carbon price forecasting. Gao and Shao [24] integrated Multivariate Variational Mode Decomposition (MVMD) with an Interval Multilayer Perceptron (iMLP) to generate accurate interval predictions. To address the challenges of uncertainty and instability, Wang et al. [25] proposed a forecasting method based on Improved Complete Ensemble Empirical Mode Decomposition with Adaptive Noise (ICEEMDAN) and an interval outlier detection technique (I-ksigma). They further investigated how different combinations of interval-valued input variables affect forecasting performance. A related approach was also reported by Wang et al. [26]. Liu et al. [27] developed a two-stage decomposition-ensemble framework. First, the interval-valued carbon price series is decomposed into multiple components. Each component is then forecasted using an Interval Multilayer Perceptron enhanced by a Sparrow Search Algorithm (SSA-iMLP). Finally, all component forecasts are reconstructed to produce the final interval prediction. Despite their effectiveness, these hybrid methods rely heavily on sophisticated parameter optimization. Moreover, they often struggle to generate reliable long-term forecasts sufficient for investment decision making.

Decomposition-based multi-scale modeling—commonly formalized as decomposition, separate modeling, and ensemble—has become a well-established paradigm for carbon price forecasting [28,29]. This approach decomposes a carbon price series into components at different scales and models each component individually to capture multi-scale characteristics. However, existing studies within this paradigm face three critical bottlenecks in long-term interval carbon price forecasting. First, generic models applied to decomposed components are often inadequate for capturing both long-term non-stationary trends and fine-grained, high-frequency fluctuations induced by market shocks. The selective state space model, known as Mamba [30], is a neural architecture designed for efficient and effective sequence modeling. It is particularly well suited to long-sequence, high-throughput applications. By incorporating selective state space models (selective SSMs), Mamba dynamically determines whether to propagate or discard information based on the input. This mechanism enhances the model’s capacity to capture long-range temporal dependencies [31]. Mamba has been successfully applied to time-series forecasting [32], speech recognition [33], and natural language processing [34]. Motivated by these advances, we propose the Temporal Trend and Fluctuation Learning (TTFL) model for interval-valued carbon price forecasting. TTFL integrates wavelet decomposition with two specialized modules: a bidirectional enhanced Mamba module for trend learning and a Mamba–attention hybrid module for fluctuation learning. In addition, we introduce an interval-valued recovery loss function that quantifies the overlap between predicted and true interval prices. This loss function is the first task-specific objective designed explicitly for interval-valued carbon price forecasting. We further embed multivariate collaborative learning throughout the entire modeling pipeline. To the best of our knowledge, this work represents the first application of an improved SSM/Mamba architecture to long-term interval carbon price forecasting. It addresses the lack of task-specific loss functions in this domain and overcomes key limitations of traditional decomposition-ensemble methods. The proposed model not only achieves superior forecasting performance but also offers stakeholders a practical tool for decision making in complex carbon trading markets. The main contributions of this work are summarized as follows:

(1) This study introduces a novel forecasting framework that decomposes interval-valued carbon price series via wavelet transformation into low-frequency and high-frequency components. These components respectively capture long-term trend evolution and short-term market fluctuations. The proposed design effectively addresses the challenges of modeling long-range dependencies and irregular fluctuation patterns inherent in complex carbon price dynamics.
(2) The Price Trend Learning (PTL) module integrates two selective state space models to capture forward and backward temporal dependencies. This bidirectional architecture enhances both the accuracy and the reliability of interval-valued carbon price forecasts. By jointly optimizing the two SSM branches, the module reduces individual model biases and captures a more complete spectrum of carbon price dynamics.
(3) We introduce an interval-valued recovery loss function that quantifies the overlap between predicted and true interval prices. This loss emphasizes trend fidelity and stabilizes the training process. It provides a standardized metric for evaluating how well forecasted intervals align with observed carbon prices, thereby improving transparency and evaluation rigor in carbon market forecasting.
(4) Extensive comparative experiments demonstrate that the proposed model achieves superior forecasting accuracy and robustness under diverse market conditions. By leveraging an attention-enhanced Mamba architecture, our method produces reliable interval predictions that account for structural uncertainties. It thus offers a practical decision-support tool for carbon trading participants and environmental policymakers.

## 2. Methods

### 2.1. Overview

**Figure 1:**
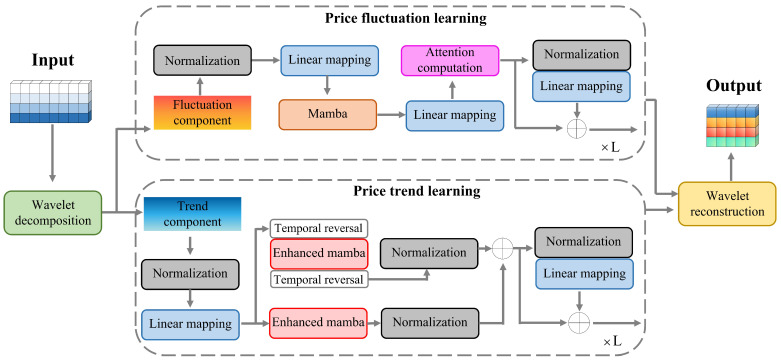
Overall framework of the proposed TTFL model. It learns features from *N* variables in the time window of $T_{1}$ and forecasts the future $T_{2}$ length for the highest and lowest carbon prices.

This study addresses a multivariate time-series forecasting task. The input comprises six market variables: opening price, closing price, highest price, lowest price, trading volume, and trading amount. The output consists of the forecasted highest and lowest prices, which together define the predicted interval-valued carbon price. Formally, we denote the input matrix as $X\in R^{N\times T_{1}}$ and the output matrix as $Y\in R^{N^{\prime }\times T_{2}}$. Here, N=6 corresponds to the six input variables, and $N^{\prime }=2$ corresponds to the two output variables (highest and lowest price). $T_{1}$ represents the lookback window length, and $T_{2}$ denotes the forecasting horizon. For long-term forecasting, we set $T_{2}>T_{1}$. Figure 1 illustrates the architecture of the proposed model. The input multivariate series is first decomposed via discrete wavelet transform (DWT) into two components: a trend component $X^{\mathrm{trend}}$ and a fluctuation component $X^{\mathrm{fluctuation}}$. The model consists of two parallel branches: the Price Trend Learning (PTL) module and the Price Fluctuation Learning (PFL) module. We describe each module in detail below.

### 2.2. Price Trend Learning

Normalization scales each feature to a fixed interval, typically [0,1]. This removes the influence of differing units and value ranges across input variables. It facilitates direct comparison across time series, prevents gradient instability caused by disparate feature scales, enhances model robustness, and improves predictive accuracy. A linear mapping (LM) layer projects the normalized time series into an embedding space. This operation produces a structured representation that enables a unified modeling framework. The transformation is defined as

(1) $$X_{nm}=LM\left(Norm\left(X^{trend}\right)\right)$$

The output of this layer has a matrix size of $N\times T_{l}$.

Mamba reduces the computational complexity of sequence modeling from quadratic to linear or logarithmic in sequence length. This ensures that performance degrades only marginally as the input horizon grows. A core component of Mamba is its selective state space mechanism, which dynamically retains or discards information based on the input content. This allows the model to focus on salient temporal features while suppressing irrelevant noise. However, the standard selective mechanism exhibits two limitations: it operates unidirectionally and favors locally proximate information. To address these issues, we propose a collaborative learning network comprising two enhanced Mamba modules. The two modules share an identical architecture but receive different inputs. The first processes the sequence in its original temporal order, while the second processes the reversed sequence. The two branches are added to the next layer. We denote the enhanced Mamba block as Ω and the temporal reversal as TR. The computation process is defined by

(2) $$Z_{f}=Norm\left(\Omega \left(X_{nm}\right)\right)$$

(3) $$Z_{r}=Norm\left(TR\left(\Omega \left(TR\left(X_{nm}\right)\right)\right)\right)$$

(4) $$Z_{o}=Z_{f}+Z_{r}$$

where $Z_{f}$ and $Z_{r}$ represent the forward and reversed enhanced Mamba, respectively. The final output of the collaborative learning network is the summation of all the enhanced Mambas and the residual input.

The normalization layer is first applied to $Z_{o}$ to promote convergence and training stability in representation learning. This layer standardizes each line of $Z_{o}$ to follow a Gaussian distribution. Subsequently, an LM layer predicts future representations using a fully connected linear transform by capturing temporal dependencies. Finally, identity mapping is used to enhance future information and effectively solve the gradient vanishing or exploding problem encountered by deep neural networks during training. The computation process is defined as follows:

(5) $$U=Z_{o}+LM\left(Norm\left(Z_{o}\right)\right)$$

This identity mapping preserves temporal information and mitigates gradient vanishing or exploding problems commonly encountered in deep networks. The output U is of the shape $N\times (T_{2}/2)$, where the reduction factor reflects the downsampling introduced by the wavelet decomposition and Mamba modules.

#### 2.2.1. Enhanced Mamba

**Figure 2:**
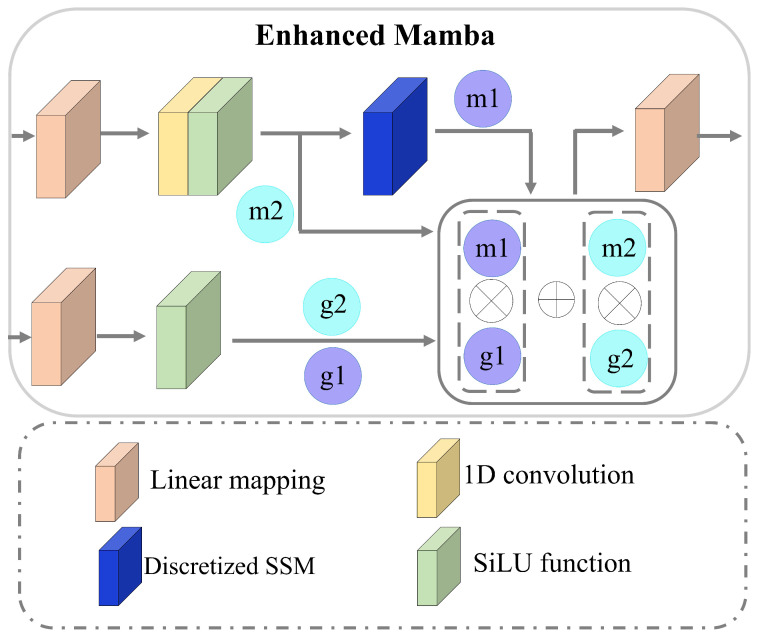
The structure of the enhanced Mamba.

Existing bidirectional state space models, such as Bi-Mamba, typically adopt a symmetric stacking design. Standard forward and backward Mamba blocks are trained independently on the original and reversed sequences. Their outputs are then concatenated or summed for feature fusion. While this design captures temporal direction complementarity, it fails to address fundamental limitations of the standard Mamba block when modeling carbon price time series—specifically, non-stationarity, sporadic volatility, and multivariate correlations. In contrast, our enhanced Mamba is a task-specific SSM architecture. It extends the standard Mamba block by integrating three key components: historical information enhancement, gated selective fusion, and bidirectional collaborative learning. Figure 2 illustrates the structure of the forward-enhanced Mamba module Ω. The module first splits the input into two parallel branches. These branches are then combined via element-wise multiplication to enhance historical information retention. A linear mapping layer first decomposes the input features $X_{nm}$ into two components. One branch is passed to a feature-selective learning mechanism; the other is directed to a gated activation function. This operation is formally expressed as

(6) $$b_{1}=LM\left(X_{nm}\right),b_{2}=LM\left(X_{nm}\right)$$

Here, $b_{1}$ and $b_{2}$ mean the first and second branches, respectively. They all have the size $N_{1}\times q$. The first branch used the combination of 1D convolutional operations and the SiLU activation function [35] to obtain local features m2, which are passed through the selective SSM to output historical information preserving features m1. The other branch directs the features $b_{2}$ through a SiLU activation function and outputs two gates: g1 and g2. The formula can be expressed by

(7) $$m2=SiLU(Conv1e\left(b_{1}\right))$$

(8) m1=SSM(m2)

(9) $$g1=SiLU(b_{2})$$

(10) $$g2=1-SiLU(b_{2})$$

After obtaining these variables, we apply multiplication and summation to output the forward Mamba features $Z_{f}$. Here is the formula:

(11) Ω=LM(m1⊗g1+m2⊗g2)

The final output of enhanced Mamba Ω returns the dimension back to $T_{l}$.

#### 2.2.2. Discretized SSM

State space models (SSMs) describe the dynamic characteristics of the system through state equations and observation equations based on the latent state of the system. It can model any cyclical process involving latent states.  SSM is used to describe these state representations and predict the state representations. There are three variables to construct first-order differential equations: input sequence I(t), latent representation H(t), and output sequence O(t). At the *t*-th time point, the hidden representation H(t) and the output O(t) can be computed by

(12) $$H{\left(t\right)}^{\prime }=A\cdot H\left(t\right)+B\cdot I\left(t\right)$$

(13) O(t)=C·H(t)

where $A\in R^{p\times p}$, $B\in R^{N\times p}$, and $C\in R^{p\times N}$. The *p* is the state dimension. The three matrices are the learning parameters. To discretize the parameters, we adopt the zero-order holding strategy and introduce the time sampling interval Δ. The new parameters are defined as

(14) $$\bar{A}=exp\left(\Delta A\right)$$

(15) $$\bar{B}={\left(\Delta A\right)}^{-1}\left(exp\left(\Delta A\right)-I\right)\cdot \Delta B$$

The discretized SSM can be redefined as

(16) $$H_{k}=\bar{A}\cdot H_{k-1}+\bar{B}\cdot I_{k}$$

(17) $$O_{k}=C\cdot H_{k}$$

**Figure 3:**
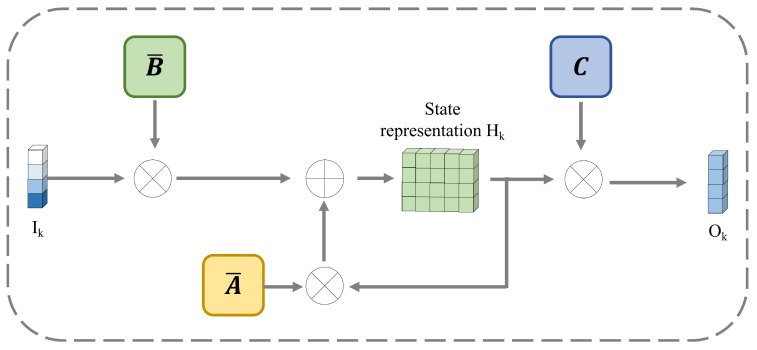
The structure of the discretized SSM.

The detailed network structure is shown in Figure 3.

### 2.3. Price Fluctuation Learning

To capture the local features of carbon price, the Price Fluctuation Learning is designed to dynamic changes. Assuming that the fluctuation component is $X^{fluctuation}$, we apply the normalization and LM to regularize the features.

(18) $$V_{1}=LM\left(Norm\left(X^{fluctuation}\right)\right)$$

The linearly mapped feature is fed into Mamba (a sequence modeling module) to capture temporal dependencies in price fluctuations. The computing formula is defined as

(19) $$V_{2}=mamba\left(V_{1}\right)$$

Then it goes through another linear mapping to adapt to the attention module. The transformed feature is processed by attention computation (denoted as Attn(·)) to capture cross-feature correlations.

(20) $$V_{3}=Attn\left(LM\left(V_{2}\right)\right)$$

The attention output is fused with intermediate features, and then passed through linear mapping and normalization to complete the local feature computing process.

(21) $$V_{4}=LM\left(Norm\left(V_{3}\right)\right)+V_{3}$$

Finally, the predicted price is computed by the wavelet reconstruction (WR), as follows:

(22) $$Y=WR(V_{4},U)$$

The final output **Y** has the size of $N^{\prime }\times T_{2}$. The key variables are defined in Table 1.

### 2.4. Loss Functions

In order to perform regression prediction for both maximum and minimum prices, we design the interval-valued recovery loss to overlap the range between predicted and true interval prices. The output $Y\in R^{N^{\prime }\times T_{2}}$ is the predicted highest and lowest prices. The loss function is defined as follows:

(23) $$L=\frac{1}{T_{2}}\sum_{i=1}^{T_{2}}\left(1-area_{i}+dist_{i}\right)$$

(24) $$area_{i}=\frac{min\left(Y_{1,i},{\dot{Y}}_{1,i}\right)-max\left(Y_{2,i},{\dot{Y}}_{2,i}\right)}{max\left(Y_{1,i},{\dot{Y}}_{1,i}\right)-min\left(Y_{2,i},{\dot{Y}}_{2,i}\right)}$$

(25) $$dist_{i}=\frac{|mean\left(Y_{1,i},Y_{2,i}\right)-mean\left({\dot{Y}}_{1,i},{\dot{Y}}_{2,i}\right)|}{max\left(Y_{1,i},{\dot{Y}}_{1,i}\right)-min\left(Y_{2,i},{\dot{Y}}_{2,i}\right)}$$

where $\dot{Y}$ and **Y** are the ground truth and predicted interval prices. Each of them has the time length $T_{2}$, and at each time point, it has two dimensions representing the upper price and the lower price. $area_{i}$ considers the degree of overlap between the two price intervals, and it falls in the range 0∼1. The larger value indicates good predicted performance. When the degree of overlap is the same, we add $dist_{i}$ to make the centers of the two price intervals as close as possible. It also ranges from 0 to 1. A small value means good predicted performance. The forecasting procedure of the CarbonMamba is illustrated in Algorithm 1.
**Algorithm 1** Forecasting process**Input:** Batch(X): ($B,N,T_{1}$) **Output:** Batch(Y): ($B,N^{\prime },T_{2}$)   
1: $X^{trend},X^{fluctuation}$ = WD(**X**)   
2: **for** *L* in PTLlayers **do**   
3:      $X_{nm}$: ($B,N,T_{l}$) ← LM(Norm(Batch($X^{trend}$)))   
4:      $Z_{f}$: ($B,N,T_{2}/2$) ←$Norm(\Omega \left(X_{nm}\right))$   
5:      $Z_{r}$: ($B,N,T_{2}/2$) ←$Norm(TR\left(\Omega \left(TR\left(X_{nm}\right)\right)\right))$   
6:      $Z_{o}$: ($B,N,T_{2}/2$) ←$Z_{f}+Z_{r}$   
7:      **U**: ($B,N,T_{2}/2$) ←$Z_{o}+Norm\left(LM\left(Z_{o}\right)\right)$   
8: **end for**   
9: **for** *L* in PFLlayers **do** 
10:      $V_{1}$: ($B,N,T_{l}$) ← LM (Norm($X^{fluctuation}$)) 
11:      $V_{2}$: ($B,N,T_{1}$) ← mamba($V_{1}$) 
12:      $V_{3}$: ($B,N,T_{2}/2$) ← Attn (LM ($V_{2}$)) 
13:      $V_{4}$: ($B,N,T_{2}/2$) ← LM (Norm ($V_{3}$)) + $V_{3}$ 
14: **end for** 
15: **Y**: ($B,N^{\prime },T_{2}$) ← WR ($V_{4}$, **U**) 
16: Return **Y**


### 2.5. Evaluation Metrics

There are five metrics used in this study to evaluate the forecasting performance: the mean absolute error (MAE), root mean squared error (RMSE), mean absolute percentage error (MAPE), coefficient of determination (R2), and mean correlation coefficient (MCC). The first three metrics have good performance when they are close to zero, and the last two metrics get better results when the values approximate one. These metrics are defined as follows:

(26) $$MAE=\frac{1}{2N_{2}}\sum_{i=1}^{N_{2}}\left(\right|Y_{1,i}-{\dot{Y}}_{1,i}\left|+\right|Y_{2,i}-{\dot{Y}}_{2,i}\left|\right)$$

(27) $$RMSE=\sqrt{\frac{\sum_{i=1}^{N_{2}}{\left(Y_{1,i}-{\dot{Y}}_{1,i}\right)}^{2}+\sum_{i=1}^{N_{2}}{\left(Y_{2,i}-{\dot{Y}}_{2,i}\right)}^{2}}{2N_{2}}}$$

(28) $$MAPE=\frac{1}{2N_{2}}\sum_{i=1}^{N_{2}}\left(\frac{|Y_{1,i}-{\dot{Y}}_{1,i}|}{{\dot{Y}}_{1,i}}+\frac{|Y_{2,i}-{\dot{Y}}_{2,i}|}{{\dot{Y}}_{2,i}}\right)$$

(29) $$MCC=\frac{PCC\left(Y_{1},{\dot{Y}}_{1}\right)+PCC\left(Y_{2},{\dot{Y}}_{2}\right)}{2}$$

(30) $$R2=1-\frac{1}{2}\frac{\sum_{i=1}^{T_{2}}{\left({\dot{Y}}_{1,i}-Y_{1,i}\right)}^{2}}{{\dot{Y}}_{1,i}-mean\left({\dot{Y}}_{1}\right)}-\frac{1}{2}\frac{\sum_{i=1}^{T_{2}}{\left({\dot{Y}}_{2,i}-Y_{2,i}\right)}^{2}}{{\dot{Y}}_{2,i}-mean\left({\dot{Y}}_{2}\right)}$$

## 3. Results

### 3.1. Data Preparation

Carbon price fluctuations reflect market expectations of allowance supply–demand balance and abatement costs. Regional heterogeneity, stemming from disparities in economic development, is a defining characteristic of China’s carbon market. To evaluate the generalizability of our model, we selected three regional pilot markets with distinct trading activity levels: two with the highest trading volumes (Hubei and Guangdong) and one with the lowest (Shanghai). Price data were obtained from the official trading platforms of the Hubei, Guangdong, and Shanghai carbon markets. For each market, we collected five daily price variables: highest price, opening price, average transaction price, closing price, and lowest price. By construction, the opening, average, and closing prices all lie within the interval defined by the daily highest and lowest prices. Our model takes these five price series over a lookback window of length $T_{1}$ as input and forecasts the highest and lowest prices over a forecasting horizon of length $T_{2}$.

The Hubei market dataset covers the period from 2 April 2014 to 29 March 2024, comprising 2292 daily observations. The Guangdong market contributes 2187 daily samples spanning 1 April 2014 to 29 March 2024. The Shanghai market yields 1475 daily records over the same period. All price series are normalized to the range [−1,1]. For model development and evaluation, we partition each dataset into training, validation, and test sets using an 80/10/10 chronological split. Table 2 summarizes the descriptive statistics for each market. To empirically justify our proposed trend–fluctuation decomposition, we conducted Augmented Dickey–Fuller (ADF) tests on the raw and wavelet-decomposed price series across all three markets. The results are reported in Table 3. For all three markets, the raw highest and lowest price series yield ADF *p*-values > 0.05, confirming their non-stationarity. This non-stationarity arises from the interplay between long-term trend evolution and short-term market shocks—dynamics that single-branch architectures cannot effectively model. The high-frequency fluctuation components exhibit strong stationarity (*p*-values < 0.05) across all markets. These components capture irregular, short-term volatility attributable to daily trading activity or transient policy events. In contrast, the low-frequency trend components remain non-stationary (*p*-values > 0.05), as they reflect the long-term, gradually evolving trajectory of carbon prices. These results collectively demonstrate that carbon price dynamics comprise two fundamentally distinct temporal structures, each requiring specialized modeling. This finding provides direct empirical support for our proposed two-branch architecture.

### 3.2. Comparison Methods

In the experiment, we adopt a causal rolling-window wavelet decomposition strategy that preserves temporal order and ensures strict separation of training, validation, and test sets. Critically, the DWT decomposition for each time window uses only historical information up to the current time step. No future data—whether from validation/test sets or future positions within the training set—contaminate the decomposition process. We first split each original carbon price series into training, validation, and test sets using a strict 80/10/10 chronological split. DWT decomposition is performed independently on each set. All decomposition hyperparameters (wavelet basis and decomposition level) are learned exclusively from the training set and then frozen. These fixed parameters are applied directly to the validation and test sets without any re-estimation or fine-tuning, thereby preventing information leakage. For the multi-step long-term interval forecasting task, we apply a causal rolling window to each of the training, validation, and test sets. Within each window, DWT decomposition is performed independently to extract the low-frequency trend and high-frequency fluctuation components. Based on the spectral properties of the training data, we select the db4 (Daubechies 4) wavelet basis with 1-level decomposition as the fixed DWT configuration.

The collaborative learning network employs a 3-layer architecture. We trained all models on identical hardware (Intel i9-13900K CPU, NVIDIA RTX 4090 GPU) and software (PyTorch 2.1.0, Python 3.9) to eliminate performance variability attributable to platform differences. We set a maximum of 800 training epochs with early stopping (patience = 50) activated when validation MAE ceases to improve. The input lookback window length is $T_{1}=14$, and the forecasting horizon is $T_{2}\in 7,14,21$. We use a batch size of 8 and an initial learning rate of 0.001. Weight decay is fixed at $1\times 10^{-5}$ to mitigate overfitting. The Adam optimizer is employed with $\beta_{1}=0.9$ and $\beta_{2}=0.999$. For the discretized SSM within the enhanced Mamba module, the hidden state dimension is set to p=128. In the PFL module, the multi-head attention mechanism uses 4 heads with a hidden dimension of 256 and a dropout rate of 0.1. This configuration captures diverse cross-feature correlations among multivariate carbon price variables while regularizing against overfitting. All hyperparameters remain fixed across the Hubei, Guangdong, and Shanghai datasets to ensure experimental consistency. For comparative evaluation, we selected five baseline methods for interval-valued carbon price forecasting.

- Multilayer Perceptron (MLP) method [36]. This model adopts a fully connected architecture with an input layer, 3 hidden layers (256 neurons per layer), and an output layer, where ReLU activation is applied to intermediate layers and no activation is used for the regression-oriented output layer. The 14 × 6 temporal input is flattened into an 84-dimensional vector, with dropout = 0.2 and weight decay = 1.0 $\times \;10^{-5}$ for overfitting mitigation. Training is implemented with the Adam optimizer ($\beta_{1}$ = 0.9, $\beta_{2}$ = 0.999) at a learning rate of 0.001, with a batch size of 8, maximum 800 epochs, and early stopping (Patience = 50) based on validation MAE.
- Time-Series Dense Encoder (TiDE) method [37]. TiDE is built on an encoder–decoder framework, where both encoder and decoder consist of 3 fully connected layers (128 neurons per layer) with GELU activation for efficient gradient propagation. It captures temporal dependencies via local time window attention (window size = 3) and maps 6-dimensional inputs to a 64-dimensional feature space for enhancement, with dropout = 0.2 and weight decay = 1.0 × $10^{-5}$ as regularization. Uniform training settings are adopted: Adam optimizer, learning rate = 0.001, batch size = 8, 800 maximum epochs, and early stopping (Patience = 50) for fair comparison.
- Gated Recurrent Unit (GRU) method [38]. The model is composed of 2 GRU layers (128 hidden dimensions per layer) and a fully connected output layer, with inter-layer dropout = 0.2 to avoid overfitting. It processes input sequences of shape (batch size, 14, 6) by iterative hidden state updating for long-term temporal dependency capture, and the output layer maps 128-dimensional hidden states to 2-dimensional predictions (highest/lowest carbon price). Training uses the Adam optimizer (learning rate = 0.001) with batch size = 8, 800 maximum epochs and early stopping (Patience = 50); gradient clipping (max norm = 5.0) is additionally applied to prevent gradient explosion.
- Temporal Convolutional Networks (TCNs) method [39]. TCN includes 3 layers of 1D causal convolutions with kernel size = 3, 128 output channels per layer and dilation factors [1,2,4] for multi-scale temporal dependency capture. Each layer is equipped with ReLU activation, LayerNorm, and residual connections (1 × 1 convolution for dimension alignment), with dropout = 0.2 and weight decay = 1.0 × $10^{-5}$ for regularization. Training follows the unified standard: Adam optimizer, learning rate = 0.001, batch size = 8, 800 maximum epochs, and early stopping (Patience = 50).
- iTransformer method [40]. This model has 3 encoder layers with 4 attention heads each and an attention hidden dimension of 256; the 6-dimensional input is mapped to a 64-dimensional embedding space, and input sequence reversal is adopted to enhance long-term dependency capture. Its feedforward network is a 2-layer fully connected structure (512 hidden dimensions) with GELU activation, combined with dropout = 0.2 and LayerNorm for regularization. Training uses the AdamW optimizer (weight decay = 1.0 $\times \;10^{-5}$) at a learning rate of 0.001, with batch size = 8, 800 maximum epochs and early stopping (Patience = 50), tailored for long-sequence carbon price forecasting.
- Skip-Timeformer method [41]. The model adopts a skip-time embedding strategy (skip interval = 2) to sample input sequences into skip-time tokens, and captures cross-skip subsequence dependencies via 3 encoder layers (4 attention heads per layer, 256 attention hidden dimensions, 512 feedforward dimensions). It fuses original and skip-time tokens through skip-time interaction conditional LayerNorm for multi-scale pattern mining, with GELU activation, dropout = 0.2, and weight decay = 1.0 $\times \;10^{-5}$ for regularization. Training uses consistent settings: Adam optimizer, learning rate = 0.001, batch size = 8, 800 maximum epochs, and early stopping (Patience = 50).
- S-Mamba method [42]. S-Mamba consists of two bidirectional Mamba layers (SSM hidden dimension = 128, state expansion factor = 2), 1 feedforward layer (256 hidden dimensions, SiLU activation), and a linear output layer. Bidirectional Mamba layers mine inter-variable global mutual information, and the input layer linearly maps 6-dimensional inputs to a 128-dimensional feature space; dropout = 0.2 and weight decay = 1.0 $\times \;10^{-5}$ are set for regularization. Training is implemented with the Adam optimizer (learning rate = 0.001) at batch size = 8, 800 maximum epochs, and early stopping (Patience = 50), utilizing the Mamba architecture’s efficiency in long-sequence modeling.

### 3.3. Forecasting Results

**Figure 4:**
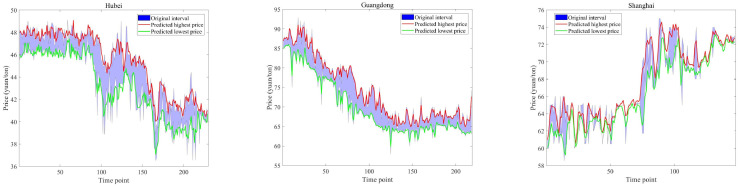
Interval forecasting of the proposed model for Hubei, Guangdong, and Shanghai markets.

**Figure 5:**
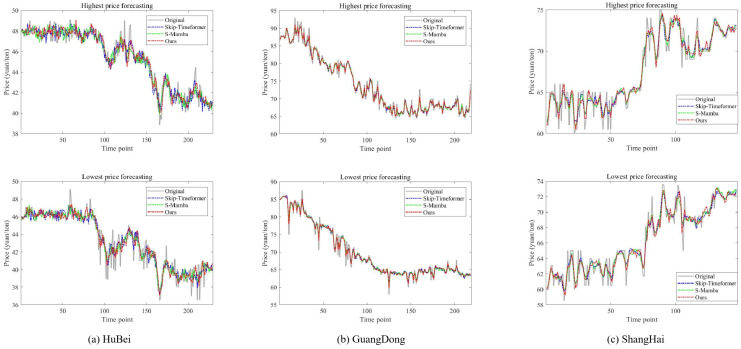
Price forecasting performance under three competing methods.

After completing the training process, Figure 4 shows the predicted interval carbon prices for the Hubei, Guangdong, and Shanghai markets. The original highest and lowest price series exhibit substantial volatility. Nevertheless, our proposed TTFL model successfully captures the underlying trajectory of interval-valued carbon prices. Figure 5 presents a comparison of price predictions from three representative methods: Skip-Timeformer, S-Mamba, and our TTFL model. While all three methods produce predictions that closely track the ground truth, our model consistently achieves closer alignment with the original price interval.

To compare with other related methods, we compute the MAE, RMSE, MAPE, MCC, and R2 for each method. Table 4, Table 5 and Table 6 show the interval price prediction performance for Hubei, Guangdong, and Shanghai markets, respectively. Furthermore, we investigate the impact of predicted length on forecasting performance. The predicted length includes 7, 14, and 21. For each method, the prediction performance drops when the time length is longer. This is because the carbon price is highly complex and non-stationary, making it hard to model long time series. For the Hubei market, our model achieves the best results with the MAE, RMSE, MAPE, MCC, and R2 values of 0.5802, 0.7760, 0.0134, 0.9636, and 0.9282, respectively. For the Guangdong market, our model achieves the best results with MAE, RMSE, MAPE, MCC, and R2 values of 0.5119, 0.6984, 0.0071, 0.9958, and 0.9916, respectively. For the Shanghai market, our model achieves the best results with MAE, RMSE, MAPE, MCC, and R2 values of 0.7116, 0.9456, 0.0108, 0.9749, and 0.9501, respectively. For each method, the longer the length of the predicted time series, the greater the prediction error.

**Figure 6:**
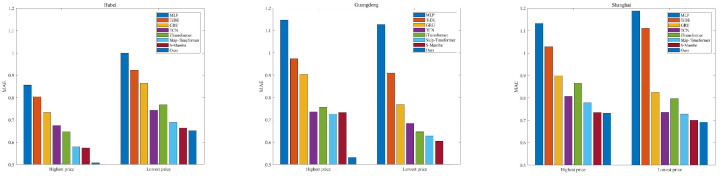
MAEs of highest and lowest price forecasting for predicted length = 7.

**Figure 7:**
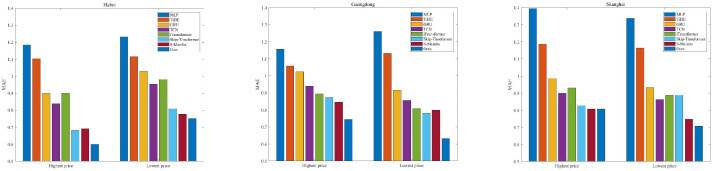
MAEs of highest and lowest price forecasting for predicted length = 14.

**Figure 8:**
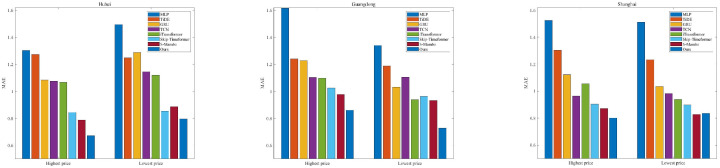
MAEs of highest and lowest price forecasting for predicted length = 21.

For the same time length, our model achieves the best forecasting performance. The MLP-based methods (MLP and TiDE) show the worst results because their linear mapping relationships cannot capture the complex nonlinear dynamics. The GRU performs relatively better than MLP-based methods since it can selectively retain and forget information. When the predicted time length is long, the GRU likely encounters the gradient vanish problem. The TCN and iTransformer both show good forecasting performance. The Skip-timeformer and S-Mamba show better forecasting performance. This indicates that they have the ability to model dynamic fluctuations and static trend features. Figure 6, Figure 7 and Figure 8 show the MAEs of highest and lowest prices forecasting for different time lengths. For the Hubei and Guangdong markets, the MAEs of highest price forecasting reach the minimum value at our method; a similar result is also observed in the lowest price forecasting experiments. For experiments with a prediction length of 7, our method also shows the best predicted MAEs except for the Shanghai market. The worst results are from the MLP method for all three markets.

### 3.4. Ablation Study

To investigate the contribution of each core module in the proposed TTFL model, we conduct an ablation study on the three datasets (Hubei, Guangdong, Shanghai). The MAE is the evaluation metric. The four variations are as follows: (1) TTFL w/o PFL, we replace PFL with PTL. (2) TTFL w/o PTL, we we replace PTL with PFL. (3) TTFL w/o attention, we replace the attention module with traditional Mamba. (4) TTFL w/o enhanced Mamba, we replace the enhanced Mamba with traditional Mamba.

Table 7 summarizes the ablation experimental results. Compared with the full TTFL model, removing the Price Fluctuation Learning module (TTFL without PFL) leads to a substantial increase in prediction error. This confirms that PFL effectively captures short-term, high-frequency volatility—a critical capability for markets characterized by frequent price fluctuations. Removing the attention mechanism (TTFL w/o attention) results in a moderate MAE rise, while omitting enhanced Mamba (TTFL w/o enhanced Mamba) causes MAE increase. These results demonstrate that the attention mechanism strengthens cross-regional feature interaction, and enhanced Mamba optimizes sequential dependency modeling—both serve as complementary backbones for the model. PFL and PTL focus on domain-specific price patterns, while the attention-enhanced Mamba framework enhances general sequence modeling capabilities, enabling the model’s state-of-the-art forecasting results. Additionally, replacing the enhanced Mamba with simple stacked standard Bi-Mamba blocks (denoted as TTFL w/o enhanced Mamba) clearly demonstrates the superiority: for the Hubei market, the MAE increases from 0.6754 (our enhanced Mamba) to 0.7109 (stacked standard Bi-Mamba); for the Guangdong market, the MAE increases from 0.6880 to 0.7156; and for the Shanghai market, the MAE increases from 0.7570 to 0.7916. The significant MAE increase in the ablation experiment directly proves that simple stacking of standard Mamba blocks leads to degraded forecasting performance, while our enhanced Mamba architecture achieves better feature extraction and trend modeling for carbon price time series.

We have conducted the Diebold–Mariano (DM) test (a standard statistical test for comparing time-series forecasting models) to assess whether the performance improvements of each core module (PFL, PTL, attention, enhanced Mamba) are statistically significant at the 95% confidence level. As shown in Table 8, the *p*-values across all markets confirm that removing the PFL branch leads to a statistically significant performance drop—this aligns with our original claim and validates that the PFL module’s ability to capture high-frequency fluctuations is critical for reducing prediction errors. Even though the MAE gap between TTFL and TTFL w/o attention appears relatively small, the DM test *p*-values are all <0.05. This proves that the attention mechanism’s enhancement of cross-feature correlations (in the PFL branch) contributes statistically meaningful improvements, rather than random fluctuations. TTFL w/o PTL and TTFL w/o enhanced Mamba: Both variants yield *p*-values < 0.05 across all markets, confirming that the PTL branch (for long-term trend modeling) and enhanced Mamba (for optimized sequential dependency capture) are indispensable for the model’s performance.

## 4. Discussion

**Figure 9:**
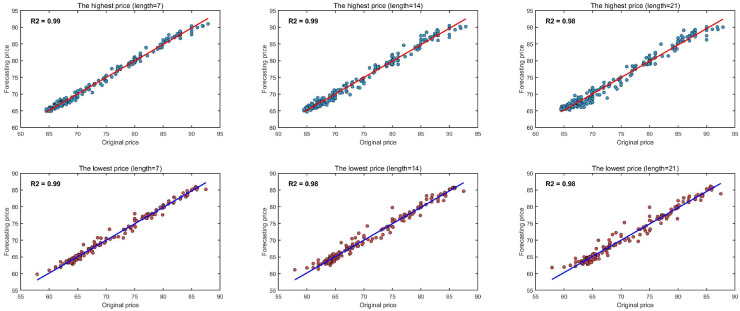
Correlation between the original and forecasting prices under different lengths.

Carbon price series across different regional markets exhibit pronounced stochasticity and non-stationarity. Among all eight compared methods, our proposed TTFL model achieves superior forecasting performance due to its ability to capture the underlying trends in interval-valued carbon prices. The model decomposes the forecasting task via wavelet transformation into two specialized branches: a trend learning branch and a fluctuation learning branch. For the low-frequency component (long-term price trends), we adopt an enhanced Mamba module. This module leverages near-linear complexity to model long-range temporal dependencies, enabling efficient and accurate extraction of long-term carbon price dynamics. For the high-frequency component (short-term fluctuations), we employ an attention mechanism. It adaptively focuses on salient fluctuation patterns and highlights temporal dependencies in price volatility. This enables precise modeling of irregular price movements driven by market supply and demand, as well as other exogenous factors. We further introduce an interval-valued recovery loss function, which significantly improves both predictive accuracy and training stability. In contrast with traditional point forecasting methods, our approach emphasizes trend prediction and provides decision makers with more comprehensive information for carbon market analysis and policy planning. Figure 9 visualizes the predicted versus true interval-valued prices for the Hubei market under three forecasting horizons ($T_{2}=7,14,21$). Each subfigure corresponds to the highest and lowest price predictions. Across all horizons, the predicted values are tightly clustered around the diagonal perfect-prediction line (y=x). The coefficient of determination ($R^{2}$) remains above 0.98 in all cases, indicating strong agreement between predictions and ground truth. As the forecasting horizon increases from 7 to 21 steps, $R^{2}$ exhibits a slight decline from 0.99 to 0.98. This degradation is consistent with the well-documented challenge of long-horizon time-series forecasting. Nevertheless, our model maintains high predictive accuracy and reliably captures both short-term and long-term price patterns.

The proposed TTFL model generates interval-valued carbon price forecasts that offer market participants quantifiable upper and lower bounds for future price movements, thereby guiding trading decisions. For industrial enterprises subject to emission reduction obligations, the lower bound of the predicted interval provides an opportunity to purchase spot allowances at favorable prices, mitigating the risk of future price increases. When predicted intervals widen, enterprises can hedge exposure using carbon future contracts. For professional traders, short-term interval predictions enable range trading strategies, while long-term interval forecasts support medium-term position establishment. The model’s high predictive accuracy enhances the reliability of arbitrage timing. The interval predictions produced by our model provide a rigorous basis for quantifying and mitigating carbon asset risks. Financial institutions can compute value-at-risk (VaR) metrics from predicted price intervals and adjust collateral haircuts for carbon pledge financing accordingly, thereby reducing credit risk exposure from price declines. Regulators and exchange operators can employ the predicted intervals as early warning thresholds. When actual prices breach the upper or lower bounds, timely interventions—such as adjusting market stability reserves or releasing additional allowances—can be triggered. Industrial enterprises can also forecast abatement cost volatility from the predicted price intervals, enabling proactive deployment of low-carbon technologies to contain cost risks. The TTFL model’s ability to precisely capture both short-term fluctuations and long-term trends provides empirical support for carbon policy design. For the development of a unified national carbon market, policymakers can design differentiated quota allocation and market stabilization mechanisms based on the interval prediction characteristics of each regional pilot. This facilitates price convergence across heterogeneous markets. When formulating long-term emission reduction targets, the model’s extended-horizon interval forecasts can be used to evaluate the effectiveness of existing policies. A sustained upward trajectory in predicted carbon prices suggests that current targets are appropriately stringent; a flat or declining trajectory would indicate the need for moderate tightening. For regional low-carbon transition planning, policymakers can coordinate the pace of industrial restructuring based on carbon price interval forecasts. This helps avoid disruptive effects on local industries from severe price volatility.

The proposed enhanced Mamba module introduces three key innovations that address fundamental limitations of existing bidirectional state space models (e.g., Bi-Mamba) and are specifically designed for modeling low-frequency trend components in carbon price time series. (1) The standard Mamba block exhibits an inherent recency bias: it preferentially emphasizes recent temporal information while failing to adequately capture long-term historical trend features critical for carbon price forecasting. Our enhanced Mamba decomposes the input features into two parallel branches via a linear mapping layer. This two-branch architecture explicitly breaks the recency bias of the standard Mamba block and jointly enhances both global historical and local temporal features. Existing bidirectional SSMs, which rely on single-branch standard Mamba blocks, cannot achieve this functionality. (2) Carbon price time series are characterized by strong non-stationarity: the relative importance of historical and local features varies with evolving market conditions. Existing bidirectional SSMs employ fixed fusion strategies—typically concatenation or summation—that lack adaptive adjustment of feature weights in response to carbon price dynamics. This rigidity leads to suboptimal modeling of non-stationary trends. Our enhanced Mamba introduces a gated activation module based on the Sigmoid Linear Unit (SiLU). This module enables adaptive, selective fusion of the two-branch features. The gated mechanism dynamically adjusts feature importance according to the degree of non-stationarity in the carbon price series. This adaptive fusion capability fundamentally differentiates our approach from existing bidirectional SSMs with fixed fusion strategies. (3) For long-term forecasting tasks, naive stacking of standard Mamba blocks—even in bidirectional configurations—is prone to gradient vanishing. Deep networks constructed in this manner fail to propagate gradient information from long-range temporal features effectively, resulting in suboptimal modeling of extended dependencies. Our enhanced Mamba addresses this limitation through two complementary designs. First, it integrates the two-branch enhanced block into a bidirectional collaborative learning framework. Second, it introduces a residual linear mapping connection between the bidirectional fusion output and the final trend feature representation. This residual connection avoids the shallow fusion characteristic of existing bidirectional SSMs and substantially mitigates gradient vanishing in deep long-term forecasting architectures.

**Figure 10:**
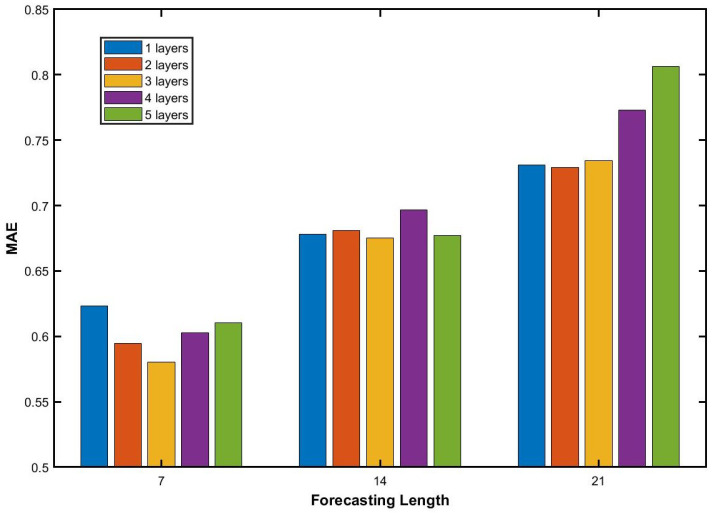
Influence of different layer numbers on the forecasting results for different forecasting lengths.

**Figure 11:**
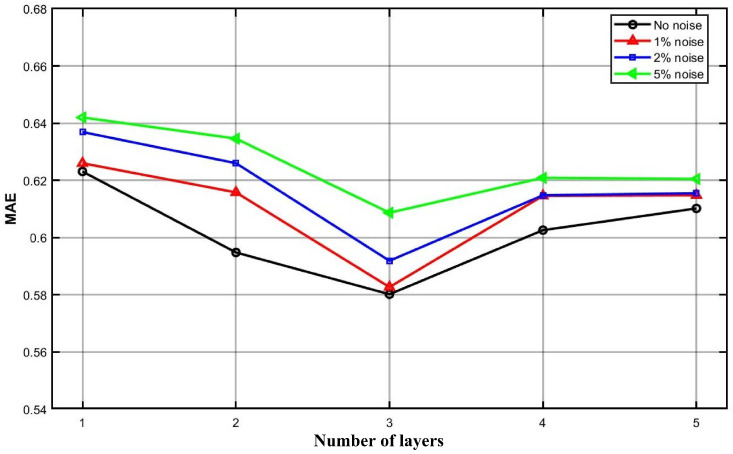
Robustness of our model under different noise levels.

The number of layers in the collaborative learning network also affects forecasting performance. We evaluate five configurations with 1 to 5 layers to assess the sensitivity of our model to architectural depth. Figure 10 presents the MAE of the TTFL model on the Hubei market under forecasting horizons of $T_{2}=7,14,21$. For all horizons, MAE increases monotonically with the forecasting horizon. The 3-layer configuration consistently achieves the lowest MAE across all horizons. At $T_{2}=7$, it outperforms all other depths. At $T_{2}=14$ and $T_{2}=21$, it remains competitive and substantially outperforms both shallower (1-layer) and deeper (4- and 5-layer) architectures. These results suggest that 3 layers strike an optimal balance between representational capacity and overfitting risk. Deeper networks introduce unnecessary complexity without performance gains, while shallower networks are insufficient to capture the temporal patterns inherent in carbon price dynamics. To evaluate the robustness of our model, we inject Gaussian noise at 3 levels (1%, 2%, and 5%) into the Hubei carbon price series. Figure 11 reports the MAE under each noise condition across different network depths. The MAE exhibits a consistent U-shaped pattern with respect to layer count: it reaches a minimum at 3 layers and increases slightly for deeper or shallower configurations. Importantly, the MAE varies only marginally across different noise levels. Even under 5% noise, the degradation relative to the noise-free baseline remains minimal. These results demonstrate that our model maintains strong robustness to data perturbations. Prediction errors remain low and consistent across both low and high noise intensities. This robustness is essential for reliable deployment on real-world carbon price series, which are characterized by inherent volatility and measurement uncertainty.

**Figure 12:**
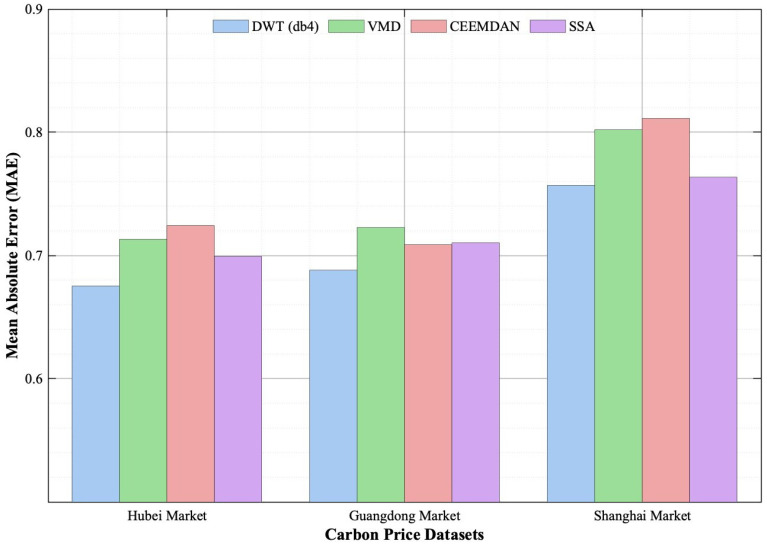
Influence of different decomposition methods on prediction performance.

Several alternative signal decomposition techniques—including Variational Mode Decomposition (VMD), Complete Ensemble Empirical Mode Decomposition with Adaptive Noise (CEEMDAN), and Singular Spectrum Analysis (SSA)—can influence interval forecasting performance. To evaluate their effectiveness within our framework, we replaced the discrete wavelet transform (DWT) in the TTFL model with each of these three methods while keeping all other modules and hyperparameters fixed. We then compared forecasting accuracies using mean absolute error (MAE) on the Hubei, Guangdong, and Shanghai datasets. Figure 12 presents the empirical results. Across all three markets, DWT consistently achieves the lowest MAE for long-horizon (14-step) interval-valued carbon price forecasting. Specifically, DWT attains MAE values of 0.6754 (Hubei), 0.6880 (Guangdong), and 0.7570 (Shanghai)—substantially lower than those obtained with VMD, CEEMDAN, or SSA. This superiority stems from DWT’s ability to cleanly separate non-stationary low-frequency trends from stationary high-frequency fluctuations. In contrast, VMD and CEEMDAN are prone to over-decomposition, while SSA often inadequately captures trend components. These decomposition characteristics align closely with the dual-branch architecture of the TTFL model. The components produced by DWT enable the Price Trend Learning (PTL) branch—built on enhanced Mamba—to model long-range temporal dependencies effectively. Simultaneously, they allow the Price Fluctuation Learning (PFL) branch—based on the Mamba–attention hybrid—to capture fine-grained volatility patterns. The consistent empirical advantage of DWT validates its suitability for the dual-scale nature of carbon price dynamics. This finding establishes a robust methodological foundation for the accuracy and robustness of the proposed TTFL model.

A notable limitation of this study is that our empirical evaluation relies exclusively on datasets from Chinese regional carbon markets. Due to differences in market mechanisms, regulatory frameworks, and external economic environments across regions, the direct generalizability of our findings to other market contexts remains to be validated. Chinese carbon markets are still in a developmental stage and exhibit lower liquidity than mature emissions trading systems such as the European Union Emissions Trading System (EU ETS). This liquidity gap contributes to higher price stickiness in Chinese markets. Consequently, the TTFL model may require targeted fine-tuning to achieve comparable performance in highly liquid international markets. Furthermore, international carbon markets are influenced by a broader set of external factors—including cross-border carbon tariffs, global energy price dynamics, and multilingual policy documents—that are not yet incorporated among the current input variables. Without accounting for these factors, the model’s generalizability to global contexts may be constrained. In future work, we plan to extend our empirical validation in several directions. First, we will expand the dataset to include key international carbon markets, beginning with the EU ETS, and compile 5–10 years of historical trading data. This will enable us to adapt the model to heterogeneous regional market characteristics. Second, we will enrich the input feature space by incorporating market-specific external factors relevant to international markets. These will include cross-border carbon allowance flow data, global energy price indices, and features extracted from international climate policy documents via natural language processing.

## 5. Conclusions

This paper introduces an enhanced attention Mamba-based framework for modeling and forecasting interval carbon prices. It integrates multiple trading prices to uncover potential relationships between them, enhancing accuracy in predicting both the lower and upper bounds of carbon price intervals. Our model divides the prediction process into high-frequency component learning and low-frequency component learning based on the non-stationarity characteristics of prices. By adding two directionally enhanced Mambas, the complementary information between prices is enhanced. Moreover, we introduce an interval-valued recovery loss metric to assess overlap between predicted and actual interval prices, prioritizing price trends and stabilizing the training process. To validate our approach, we conducted experiments on three regional markets to compute five metrics to evaluate the forecasting performance. Compared with the competing methods, our model achieves the best results with MAE values of 0.5802, 0.5119, and 0.7116 for the Hubei, Guangdong, and Shanghai markets, respectively. The proposed approach is an effective and novel approach to forecasting interval carbon prices for market trading. It can provide information support for policy formulation and investment transactions in the carbon trading market.

## Figures and Tables

**Table 1:** Definition and tensor dimension of key variables.

Variable	Physical Interpretation	Tensor Dimension
*X*	Input multivariate carbon price time series	$R^{N\times T_{1}}$
*N*	Number of input variables (6: open/close/high/low price, volume, amount)	Scalar
$T_{1}$	Input time window length	Scalar
$T_{2}$	Future forecasting length	Scalar
$N^{\prime }$	Number of output variables (2: predicted high/low price)	Scalar
$X^{trend}$	Low-frequency trend component (DWT decomposition)	$R^{N\times T_{1}/2}$
$X^{fluctuation}$	High-frequency fluctuation component (DWT decomposition)	$R^{N\times T_{1}/2}$
$X_{nm}$	Normalized and linearly mapped trend features	$R^{N\times T_{1}}$
$Z_{f}$	Forward enhanced Mamba output (normalized)	$R^{N\times T_{2}/2}$
$Z_{r}$	Reversed enhanced Mamba output (normalized)	$R^{N\times T_{2}/2}$
$Z_{o}$	Fusion of $Z_{f}$ and $Z_{r}$	$R^{N\times T_{2}/2}$
*U*	Final trend feature (residual connection)	$R^{N\times T_{2}/2}$
$V_{1}$	Normalized and linearly mapped fluctuation features	$R^{N\times T_{1}}$
$V_{2}$	Mamba output for fluctuation features	$R^{N\times T_{1}}$
$V_{3}$	Attention output for cross-feature correlation	$R^{N\times T_{2}/2}$
$V_{4}$	Final fluctuation feature (residual fusion)	$R^{N\times T_{2}/2}$
*Y*	Final predicted interval carbon price	$R^{N^{\prime }\times T_{2}}$

**Table 2:** Details of the trading prices (yuan/ton) from Hubei, Guangdong, and Shanghai.

Markets	Price	Number (Train:Val:Test)	Min	Max	Mean	Median	Std
Hubei	HIGH	1834:229:229	10.07	63.48	30.49	28.16	11.71
	OPEN		10.07	61.48	29.58	27.09	11.34
	TRAN		10.07	61.89	29.39	27.37	11.27
	CLOSE		10.07	61.48	29.64	27.70	11.32
	LOW		10.07	58.50	28.72	26.38	11.04
Guangdong	HIGH	1751:218:218	8.10	97.98	38.11	28.00	25.58
	OPEN		8.10	95.26	37.00	27.55	24.81
	TRAN		7.57	95.26	36.78	27.50	24.73
	CLOSE		8.10	95.26	37.01	27.54	24.83
	LOW		7.29	89.99	36.18	27.02	24.45
Shanghai	HIGH	1181:147:147	4.50	75.00	41.48	40.00	15.96
	OPEN		4.10	75.00	40.93	39.50	15.87
	TRAN		4.20	74.71	40.98	39.50	15.84
	CLOSE		4.20	74.71	41.01	39.60	15.81
	LOW		4.00	73.59	40.62	39.20	15.75

**Table 3:** Data stationarity test before and after wavelet decomposition (*p*-value).

	Original	Trend Component	Fluctuation Component
Hubei highest price	0.3433	0.2937	0.0013
Hubei lowest price	0.2640	0.1363	0.0020
Guangdong highest price	0.9901	0.9956	0.0012
Guangdong lowest price	0.9627	0.9741	0.0017
Shanghai highest price	0.7033	0.6688	0.0022
Shanghai lowest price	0.5989	0.6238	0.0011

**Table 4:** Forecasting results of Hubei province using different methods.

Methods	Forecasting Length	MAE	RMSE	MAPE	MCC	R2
MLP	7	0.9275	1.1467	0.0215	0.9478	0.8442
	14	1.2072	1.5173	0.0278	0.9102	0.7260
	21	1.3987	1.7412	0.0322	0.8867	0.6407
TiDE	7	0.8637	1.0924	0.0200	0.9452	0.8586
	14	1.1088	1.3739	0.0255	0.9129	0.7757
	21	1.2603	1.5739	0.0289	0.8847	0.7033
GRU	7	0.7992	1.0244	0.0184	0.9376	0.8755
	14	0.9640	1.2018	0.0221	0.9149	0.8288
	21	1.1865	1.5254	0.0273	0.8739	0.7242
TCN	7	0.7091	0.9297	0.0164	0.9548	0.8975
	14	0.8959	1.1663	0.0207	0.9246	0.8388
	21	1.1106	1.4348	0.0254	0.8805	0.7558
iTransformer	7	0.7082	0.9447	0.0164	0.9458	0.8941
	14	0.9404	1.1919	0.0216	0.9138	0.8316
	21	1.0929	1.4002	0.0251	0.8828	0.7676
Skip-Timeformer	7	0.6342	0.8248	0.0147	0.9589	0.9193
	14	0.7453	0.9453	0.0171	0.9465	0.8940
	21	0.8485	1.0982	0.0194	0.9275	0.8561
S-Mamba	7	0.6195	0.8241	0.0143	0.9588	0.9194
	14	0.7328	0.9473	0.0169	0.9458	0.8936
	21	0.8369	1.0878	0.0192	0.9312	0.8597
Ours	7	0.5802	0.7760	0.0134	0.9636	0.9282
	14	0.6754	0.8958	0.0156	0.9515	0.9047
	21	0.7343	0.9526	0.0169	0.9449	0.8925

**Table 5:** Forecasting results of Guangdong province using different methods.

Methods	Forecasting Length	MAE	RMSE	MAPE	MCC	R2
MLP	7	1.1367	1.4553	0.0160	0.9818	0.9638
	14	1.2083	1.5508	0.0170	0.9792	0.9588
	21	1.4769	1.8574	0.0206	0.9710	0.9414
TiDE	7	0.9421	1.1619	0.0133	0.9885	0.9769
	14	1.0945	1.4031	0.0154	0.9834	0.9661
	21	1.2140	1.5453	0.0170	0.9798	0.9593
GRU	7	0.8368	1.1633	0.0116	0.9884	0.9768
	14	0.9697	1.3045	0.0134	0.9855	0.9710
	21	1.1293	1.5040	0.0158	0.9807	0.9616
TCN	7	0.7107	1.0076	0.0099	0.9912	0.9825
	14	0.8969	1.1771	0.0125	0.9888	0.9764
	21	1.1043	1.4354	0.0154	0.9827	0.9647
iTransformer	7	0.7011	0.9887	0.0097	0.9916	0.9833
	14	0.8510	1.1877	0.0118	0.9879	0.9758
	21	1.0178	1.3576	0.0141	0.9842	0.9686
Skip-Timeformer	7	0.6775	0.9682	0.0094	0.9920	0.9840
	14	0.8263	1.1178	0.0115	0.9893	0.9787
	21	0.9935	1.2878	0.0138	0.9859	0.9718
S-Mamba	7	0.6687	0.9537	0.0092	0.9922	0.9845
	14	0.8220	1.1064	0.0114	0.9895	0.9790
	21	0.9547	1.2311	0.0133	0.9870	0.9741
Ours	7	0.5119	0.6984	0.0071	0.9958	0.9916
	14	0.6880	0.9661	0.0095	0.9920	0.9841
	21	0.7940	1.1233	0.0110	0.9892	0.9785

**Table 6:** Forecasting results of Shanghai using different methods.

Methods	Length	MAE	RMSE	MAPE	MCC	R2
MLP	7	1.1608	1.4606	0.0174	0.9406	0.8806
	14	1.3663	1.6438	0.0205	0.9261	0.8493
	21	1.5181	1.8836	0.0227	0.9056	0.8020
TiDE	7	1.0695	1.3214	0.0160	0.9523	0.9021
	14	1.1763	1.4872	0.0177	0.9401	0.8767
	21	1.2686	1.5714	0.0190	0.9320	0.8623
GRU	7	0.8622	1.1274	0.0131	0.9641	0.9291
	14	0.9598	1.2393	0.0145	0.9570	0.9144
	21	1.0774	1.3614	0.0162	0.9492	0.8966
TCN	7	0.7720	1.0332	0.0117	0.9701	0.9405
	14	0.8819	1.1138	0.0133	0.9660	0.9308
	21	0.9728	1.2479	0.0146	0.9581	0.9131
iTransformer	7	0.8313	1.0781	0.0126	0.9674	0.9352
	14	0.9091	1.1857	0.0137	0.9603	0.9216
	21	0.9969	1.2662	0.0150	0.9547	0.9106
Skip-Timeformer	7	0.7534	1.0049	0.0114	0.9716	0.9437
	14	0.8574	1.1060	0.0130	0.9657	0.9317
	21	0.9007	1.1784	0.0136	0.9612	0.9226
S-Mamba	7	0.7174	0.9743	0.0109	0.9734	0.9471
	14	0.7765	1.0554	0.0117	0.9687	0.9379
	21	0.8491	1.1001	0.0128	0.9661	0.9325
Ours	7	0.7116	0.9456	0.0108	0.9749	0.9501
	14	0.7570	0.9893	0.0114	0.9729	0.9453
	21	0.8181	1.0778	0.0123	0.9677	0.9352

**Table 7:** Influence of different modules on forecasting results.

Model	MAE of Hubei	MAE of Guangdong	MAE of Shanghai
TTFL w/o PFL	0.7521	0.7507	0.8441
TTFL w/o PTL	0.7633	0.7643	0.8506
TTFL w/o attention	0.6937	0.7031	0.7832
TTFL w/o enhanced Mamba	0.7109	0.7156	0.7916
TTFL	0.6754	0.6880	0.7570

**Table 8:** Diebold–Mariano test on the ablation studies (*p*-value).

Ablated Variant	Hubei	Guangdong	Shanghai
TTFL w/o PFL	0.002	0.001	0.003
TTFL w/o PTL	0.004	0.002	0.005
TTFL w/o attention	0.018	0.023	0.029
TTFL w/o enhanced Mamba	0.012	0.015	0.021

## Data Availability

Data Availability Statement: Dataset available on request from the corresponding authors.
